# Evaluation of All-Cause and Cause-Specific Mortality by Race and Ethnicity Among Pregnant and Recently Pregnant Women in the US, 2019 to 2020

**DOI:** 10.1001/jamanetworkopen.2022.53280

**Published:** 2023-01-03

**Authors:** Jeffrey T. Howard, Jessica K. Perrotte, Caleb Leong, Timothy J. Grigsby, Krista J. Howard

**Affiliations:** Department of Public Health, University of Texas, San Antonio (J. T. Howard, Leong); Department of Psychology, Texas State University, San Marcos (Perrotte, K. J. Howard); Department of Social and Behavioral Health, University of Nevada, Las Vegas (Grigsby).

## Introduction

Research has suggested trends of worsening maternal health associated with depression,^1^ substance use,^2^ and severe maternal morbidity^3^ in the US over the past decade. All-cause and drug/alcohol poisoning mortality rates for pregnant and recently pregnant women also increased in the US from 2015 to 2019.^4^ We examined all-cause and cause-specific mortality rates among pregnant and recently pregnant women from 2019 to 2020 and compared mortality rates by race and ethnicity.

## Methods

Because this cross-sectional study used deidentified data, the University of Texas at San Antonio Institutional Review Board deemed it exempt from review, and informed consent was waived. The study followed the STROBE reporting guideline.

Deidentified Multiple Cause of Death files were obtained from the National Center for Health Statistics (NCHS; 2019–2020).^5^ Live birth counts were obtained from the US Centers for Disease Control and Prevention WONDER database.^6^ Recently pregnant women were defined as (1) pregnant at time of death or (2) died within 1 year of pregnancy end.^4^ Female biological sex was determined from death certificates. Death and live birth counts were aggregated by year (2019 and 2020), age group (10–14, 15–24, 25–34, 35–44, or 45–54 years), and race and ethnicity (as defined by WONDER and NCHS^5^ and obtained from death certificates). The *International Statistical Classification of Diseases, Tenth Revision* codes and manner of death indicated causes of death as pregnancy associated, accidental drug poisoning, motor vehicle collision, homicide, or suicide (eTable in Supplement 1).

Mortality rates per 100 000 live births were estimated using Poisson regression models with denominators as live births with 95% CIs. Mortality rate ratios (MRRs [95% CIs]) were used to compare total mortality rates between 2019 and 2020 and to compare racial and ethnic groups. *P* < .05 (2-sided) was considered statistically significant. Data were analyzed between October 4 and October 8, 2022, using R, version 4.2.1 (R Foundation for Statistical Computing).

## Results

Of 4535 total deaths from 2019 to 2020, 2904 (64%) were women aged 34 years or younger. With regard to race and ethnicity, 107 women (2.4%) were American Indian or Alaska Native, 127 (2.8%) were Asian or Pacific Islander, 671 (14.8%) were Hispanic, 1276 (28.1%) were non-Hispanic Black, 2291 (50.5%) were non-Hispanic White, and 63 (1.4%) were multiple races or ethnicities. The all-cause mortality rate for recently pregnant women increased by 29% (MRR, 1.29 [1.21–1.37]; *P* < .001) from 53.9 to 69.6 per 100 000 live births (Table 1). Mortality rates increased by 22% (MRR, 1.22 [1.12–1.32]) from 27.5 to 33.6 per 100 000 live births for pregnancy-associated causes and by 36% (1.36 [1.24–1.48]; *P* < .001) from 26.4 to 36.0 per 100 000 live births for nonpregnancy causes. Mortality rates increased significantly for drug poisoning (MRR, 1.42 [1.22–1.63]; *P* < .001), motor vehicle collision (1.31 [1.02–1.58]; *P* = .007), and homicide (1.33 [1.03–1.60]; *P* = .01). Suicide mortality rates did not increase.

Compared with non-Hispanic White women, American Indian or Alaska Native women had significantly higher mortality rates across all causes of death (Table 2). Non-Hispanic Black women had significantly higher mortality rates for all causes except drug poisoning and suicide. Hispanic women had lower mortality rates for causes including all, all nonpregnancy, drug poisoning, motor vehicle collision, and suicide. Asian or Pacific Islander women had lower mortality rates across all causes. Multiracial women had a higher mortality rate for homicide.

## Discussion

In this cross-sectional study, mortality rates among recently pregnant women increased across all causes of death except suicide from 2019 to 2020. Pregnancy-associated causes were the leading cause of death, followed by drug poisoning. Limitations of this study include the potential for misclassification of causes of death and inaccuracies in pregnancy checkbox data.

Racial and ethnic disparities in mortality among recently pregnant women were evident by cause of death. Compared with non-Hispanic White women, mortality rates were 3- to 5-fold higher among American Indian or Alaska Native women for every cause, including suicide. Likewise, these findings suggest that non-Hispanic Black women experienced significantly higher mortality rates across causes, with the highest rates for homicide. Enhanced surveillance and intervention for these vulnerable groups may be warranted.

## Supplementary Material

Supplement1 SUPPLEMENT 1.
**eTable.** *International Statistical Classification of Diseases, Tenth Revision* Codes and Manner of Death to Determine Each Cause of Death

Supplement 2 SUPPLEMENT 2.
Data Sharing Statement

## Figures and Tables

**Table 1.** Number of Deaths, Age-Adjusted Mortality Rates per 100 000 Live Births, and Mortality Rate Ratios for Deaths From All Causes and Specific Causes Among Pregnant and Recently Pregnant Women, 2019 to 2020

Cause of death	2019	2020	Mortality rate ratio (95% CI)^a^	*P* value
All causes				
No. of deaths	2019	2516	1.29 (1.21–1.37)	<.001
Mortality rate (95% CI)	53.9 (50.3–57.5)	69.6 (65.3–74.0)		
Pregnancy associated				
No. of deaths	1030	1214	1.22 (1.12–1.32)	<.001
Mortality rate (95% CI)	27.5 (25.0–30.0)	33.6 (30.9–36.3)		
All nonpregnancy				
No. of deaths	989	1302	1.36 (1.24–1.48)	<.001
Mortality rate (95% CI)	26.4 (23.9–28.9)	36.0 (32.8–39.2)		
Drug poisoning				
No. of deaths	321	442	1.42 (1.22–1.63)	<.001
Mortality rate (95% CI)	8.6 (7.3–9.9)	12.2 (10.5–14.0)		
Motor vehicle collision				
No. of deaths	167	213	1.31 (1.02–1.58)	.007
Mortality rate (95% CI)	4.5 (3.5–5.4)	5.9 (4.6–7.1)		
Homicide				
No. of deaths	149	190	1.33 (1.03–1.60)	.01
Mortality rate (95% CI)	4.0 (3.1–4.8)	5.3 (4.1–6.4)		
Suicide				
No. of deaths	115	110	0.97 (0.71–1.26)	.95
Mortality rate (95% CI)	3.1 (2.2–3.9)	3.0 (2.2–3.9)		

a Reflects the comparison of 2019 mortality rates with 2020 mortality rates.

**Table 2.** Age- and Time Period–Adjusted Mortality Rate Ratios for Cause of Death by Race and Ethnicity, 2019 to 2020

Cause of death by race and ethnicity	Mortality rate ratio (95% CI)^a^	*P* value
All causes		
American Indian or Alaska Native	3.49 (2.85–4.22)	<.001
Asian or Pacific Islander	0.25 (0.29–0.42)	<.001
Hispanic	0.65 (0.60–0.71)	<.001
Non-Hispanic Black	1.92 (1.79–2.05)	<.001
Non-Hispanic White	1 [Reference]	NA
Multiple races or ethnicities	0.65 (0.50–0.82)	<.001
Pregnancy associated		
American Indian or Alaska Native	3.14 (2.24–4.27)	<.001
Asian or Pacific Islander	0.66 (0.53–0.81)	<.001
Hispanic	0.94 (0.83–1.05)	.30
Non-Hispanic Black	2.77 (2.51–3.05)	<.001
Non-Hispanic White	1 [Reference]	NA
Multiple races or ethnicities	0.59 (0.38–0.87)	.01
All nonpregnancy		
American Indian or Alaska Native	3.73 (2.88–4.75)	<.001
Asian or Pacific Islander	0.14 (0.09–0.19)	<.001
Hispanic	0.45 (0.39–0.51)	<.001
Non-Hispanic Black	1.34 (1.21–1.48)	<.001
Non-Hispanic White	1 [Reference]	NA
Multiple races or ethnicities	0.67 (0.48–0.91)	.01
Drug poisoning		
American Indian or Alaska Native	3.07 (2.02–4.66)	<.001
Asian or Pacific Islander	0.07 (0.03–0.16)	<.001
Hispanic	0.22 (0.16–0.29)	<.001
Non-Hispanic Black	0.84 (0.69–1.02)	.07
Non-Hispanic White	1 [Reference]	NA
Multiple races or ethnicities	0.65 (0.39–1.08)	.10
Motor vehicle collision		
American Indian or Alaska Native	5.25 (3.12–8.28)	<.001
Asian or Pacific Islander	0.13 (0.03–0.35)	<.001
Hispanic	0.52 (0.38–0.70)	<.001
Non-Hispanic Black	1.54 (1.21–1.96)	<.001
Non-Hispanic White	1 [Reference]	NA
Multiple races or ethnicities	0.49 (0.17–1.07)	.11
Homicide		
American Indian or Alaska Native	2.88 (1.17–7.08)	.02
Asian or Pacific Islander	0.28 (0.09–0.87)	.03
Hispanic	0.99 (0.70–1.39)	.92
Non-Hispanic Black	5.30 (4.12–6.81)	<.001
Non-Hispanic White	1 [Reference]	NA
Multiple races or ethnicities	2.33 (1.28–4.24)	.006
Suicide		
American Indian or Alaska Native	4.07 (2.00–7.35)	<.001
Asian or Pacific Islander	0.31 (0.12–0.64)	.005
Hispanic	0.34 (0.22–0.51)	<.001
Non-Hispanic Black	0.46 (0.29–0.71)	<.001
Non-Hispanic White	1 [Reference]	NA
Multiple races or ethnicities	0.81 (0.32–1.68)	.60

Abbreviation: NA, not applicable.

a Reflects the comparison of mortality rates among women from racial and ethnic minority groups with mortality rates among non-Hispanic White women for each cause.
