# Supplementary material for: Evaluation of All-Cause and Cause-Specific Mortality by Race and Ethnicity Among Pregnant and Recently Pregnant Women in the US, 2019 to 2020
Source: JAMA Netw Open. Author manuscript; Available in PMC 2023 May 18. (PMC10193819; doi:10.1001/jamanetworkopen.2022.53280)
Supplement: Supplement1 — SUPPLEMENT 1. eTable. International Statistical Classification of Diseases, Tenth Revision Codes and Manner of Death to Determine Each Cause of Death [file NIHMS1882522-supplement-Supplement1.pdf]

Supplementary Online Content

Howard JT, Perrotte JK, Leong C, Grigsby TJ, Howard KJ. Evaluation of all-cause and cause-specific mortality by race and ethnicity among pregnant and recently pregnant women in the US, 2019 to 2020. *JAMA Netw Open*. 2023;6(1):e2253280. doi:10.1001/jamanetworkopen.2022.53280

eTable. *International Statistical Classification of Diseases, Tenth Revision* Codes and Manner of Death to Determine Each Cause of Death

This supplementary material has been provided by the authors to give readers additional information about their work.

© 2023 Howard JT et al. *JAMA Network Open*.

eTable. *International Statistical Classification of Diseases, Tenth Revision* Codes and Manner of Death to Determine Each Cause of Death

**Cause or Manner of Death**	**ICD-10 Codes or Manner of Death**
Pregnancy-Associated	Determined by ICD-10 codes: O00-O99
Non-Pregnancy	Determined by ICD-10 codes: All codes except O00-O99
Drug Poisoning	Determined by ICD-10 codes: X40-X44
Motor Vehicle Accidents	Determined by ICD-10 codes: V02-V04,V09.0,V09.2,V12-V14,V19.0-V19.2,V19.4-V19.6,V20-V79,V80.3-V80.5,V81.0-V81.1,V82.0-V82.1,V83-V86,V87.0-V87.8,V88.0-V88.8,V89.0,V89.2
Homicide	Determined by manner of death: Homicide
Suicide	Determined by manner of death: Suicide

© 2023 Howard JT et al. *JAMA Network Open*.
