# Supplementary material for: Evaluation of All-Cause and Cause-Specific Mortality by Race and Ethnicity Among Pregnant and Recently Pregnant Women in the US, 2019 to 2020
Source: JAMA Netw Open. Author manuscript; Available in PMC 2023 May 18. (PMC10193819; doi:10.1001/jamanetworkopen.2022.53280)
Supplement: Supplement 2 — Data Sharing Statement [file NIHMS1882522-supplement-Supplement_2.pdf]

Data Sharing Statement

Howard. Evaluation of All-Cause and Cause-Specific Mortality by Race and Ethnicity Among Pregnant and Recently Pregnant Women in the US, 2019 to 2020. *JAMA Netw Open*. Published January 27, 2023. doi:10.1001/jamanetworkopen.2022.53280

Data
Data available: No

Additional Information
Explanation for why data not available: The data used for the analysis are restricted-use data from the National Center for Health Statistics, and can only be accessed by researchers with signed data use agreements.
